# High Fat Diet Attenuates the Anticontractile Activity of Aortic PVAT via a Mechanism Involving AMPK and Reduced Adiponectin Secretion

**DOI:** 10.3389/fphys.2018.00051

**Published:** 2018-02-09

**Authors:** Tarek A. M. Almabrouk, Anna D. White, Azizah B. Ugusman, Dominik S. Skiba, Omar J. Katwan, Husam Alganga, Tomasz J. Guzik, Rhian M. Touyz, Ian P. Salt, Simon Kennedy

**Affiliations:** ^1^Institute of Cardiovascular and Medical Sciences, College of Medical, Veterinary and Life Sciences, University of Glasgow, Glasgow, United Kingdom; ^2^Medical School, University of Zawia, Zawia, Libya; ^3^Department of Physiology, National University of Malaysia Medical Centre, Kuala Lumpur, Malaysia; ^4^Jagiellonian University College of Medicine, Krakow, Poland; ^5^Department of Biochemistry, College of Medicine, University of Diyala, Baqubah, Iraq

**Keywords:** perivascular adipose tissue, AMPK, high-fat diet, adiponectin, anticontractile effect

## Abstract

**Background and aim:** Perivascular adipose tissue (PVAT) positively regulates vascular function through production of factors such as adiponectin but this effect is attenuated in obesity. The enzyme AMP-activated protein kinase (AMPK) is present in PVAT and is implicated in mediating the vascular effects of adiponectin. In this study, we investigated the effect of an obesogenic high fat diet (HFD) on aortic PVAT and whether any changes involved AMPK.

**Methods:** Wild type Sv129 (WT) and AMPKα1 knockout (KO) mice aged 8 weeks were fed normal diet (ND) or HFD (42% kcal fat) for 12 weeks. Adiponectin production by PVAT was assessed by ELISA and AMPK expression studied using immunoblotting. Macrophages in PVAT were identified using immunohistochemistry and markers of M1 and M2 macrophage subtypes evaluated using real time-qPCR. Vascular responses were measured in endothelium-denuded aortic rings with or without attached PVAT. Carotid wire injury was performed and PVAT inflammation studied 7 days later.

**Key results:** Aortic PVAT from KO and WT mice was morphologically indistinct but KO PVAT had more infiltrating macrophages. HFD caused an increased infiltration of macrophages in WT mice with increased expression of the M1 macrophage markers *Nos2* and *Il1b* and the M2 marker *Chil3*. In WT mice, HFD reduced the anticontractile effect of PVAT as well as reducing adiponectin secretion and AMPK phosphorylation. PVAT from KO mice on ND had significantly reduced adiponectin secretion and no anticontractile effect and feeding HFD did not alter this. Wire injury induced macrophage infiltration of PVAT but did not cause further infiltration in KO mice.

**Conclusions:** High-fat diet causes an inflammatory infiltrate, reduced AMPK phosphorylation and attenuates the anticontractile effect of murine aortic PVAT. Mice lacking AMPKα1 phenocopy many of the changes in wild-type aortic PVAT after HFD, suggesting that AMPK may protect the vessel against deleterious changes in response to HFD.

## Introduction

Obesity is an independent risk factor for the development of cardiovascular diseases including coronary artery disease, hypertension, atherosclerosis, and heart failure (Poirier et al., 2006). It has been reported that the risk of cardiovascular disease is four times higher in obese than normal weight people (Manson et al., 1995). Understanding the correlation between obesity and cardiovascular risk has focussed on studying the effect of changes in particular fat depots throughout the body and in this regard, the perivascular adipose tissue (PVAT), which surrounds most blood vessels and regulates vascular function, would appear to be of particular importance. PVAT is an endocrine tissue that produces many active molecules termed adipocytokines (Almabrouk et al., 2014) and in healthy subjects, PVAT exhibits an anticontractile effect via release of PVAT-derived relaxing factors (Aghamohammadzadeh et al., 2012). Studies have shown that PVAT attenuates vascular contraction in multiple vascular beds including coronary vessels (Aghamohammadzadeh et al., 2012), rat aorta (Löhn et al., 2002; Dubrovska et al., 2004), and rat mesenteric arteries (Verlohren et al., 2004) and there are multiple PVAT-derived agents which have been proposed to underlie this anticontractile effect including adiponectin (Fesus et al., 2007), NO (Gil-Ortega et al., 2014) and H_2_O_2_ (Gao et al., 2007).

As obesity is associated with an increased PVAT mass, it would be intuitive to expect an increased anticontractile effect of the PVAT due to enhanced release of PVAT-derived relaxing factors. However, obesity triggers structural and functional changes in PVAT which contribute to a loss or attenuation of the anticontractile effect, which in similarity to *endothelial dysfunction* has been termed *PVAT dysfunction* (Guzik et al., 2006). This may be by virtue of increased oxidative stress (Ketonen et al., 2010; Rebolledo et al., 2010; Gil-Ortega et al., 2014), hypoxia and inflammation (Greenstein et al., 2009) within the adipose tissue leading to dysfunctional adipokine release (Maenhaut et al., 2010; Gu and Xu, 2013). Although it has been shown that the anti-contractile activity of PVAT is attenuated in obese patients and animal models (Greenstein et al., 2009), the underlying mechanism of PVAT dysfunction remains elusive.

The enzyme AMP-activated protein kinase (AMPK) maintains energy homoeostasis (Carling et al., 2011) and is involved in regulation of glucose, lipid, and protein metabolism (Hardie, 2011). AMPK is activated by reduced cellular energy charge, such as that occurring in hypoxia, hypoglycaemia, and ischaemia, leading to increased phosphorylation of the catalytic AMPKα subunit at Thr172. Activated AMPK subsequently phosphorylates a number of metabolic enzymes leading to normalization of ATP levels (Bijland et al., 2013; Salt and Hardie, 2017). In addition to the well-characterized metabolic actions of AMPK, it is increasingly clear that AMPK plays an important role in the maintenance of vascular health (Salt and Hardie, 2017). Interestingly, in adipose tissue the activity of AMPK is diminished in obesity and metabolic syndrome (Ruderman et al., 2013) while in fat-fed rats, the AMPK/mTOR pathway may contribute to PVAT-mediated vascular dysfunction and remodeling (Ma et al., 2010). Adiponectin, the most abundant adipokine generated by PVAT (Fesus et al., 2007), may exert its anticontractile effect through hyperpolarisation of vascular smooth muscle cells (VSMCs) via AMPK (Weston et al., 2013), and in HFD-fed obese rats, adiponectin improves endothelial dysfunction in the aorta via AMPK activation and eNOS phosphorylation (Deng et al., 2010). Thus, reduced AMPK activity or expression in obesity could underlie the loss of the anticontractile effect of PVAT. Indeed, in a previous study, we demonstrated that aortic PVAT from mice globally deficient in AMPKα1 did not exert an anticontractile effect and this correlated with reduced adiponectin secretion (Almabrouk et al., 2017). However, whether AMPK reduction is also affecting adiponectin release from PVAT in HFD-fed animals remains unclear.

The effect of diet-induced obesity on AMPK has most frequently been studied at the level of the endothelium and VSMCs (Ma et al., 2009, 2010). These studies have yielded consistent results, showing that AMPK acts as a protective mechanism against diet-induced obesity. For example, fructose-fed rats exhibit dysfunction of adipocytokine expression in PVAT and loss of endothelium-dependent vasodilation which can be reversed by activating AMPK (Sun et al., 2014). In fat-fed rats, treatment with a steroid sapogenin (diosgenin) enhanced AMPK phosphorylation in PVAT and reduced inflammatory markers, an effect reversed by AMPK knockdown using siRNA (Chen et al., 2016). A recent study by Zaborska et al. demonstrated that the male offspring of female rats fed a HFD during pregnancy and lactation had dysfunctional mesenteric PVAT which correlated with reduced AMPK activity in the PVAT and reduced NO bioavailability (Zaborska et al., 2016). In a further study by the same group it was found that O-GlcNAcylation was the likely cause of reduced AMPK activity and that the anticontractile effect of PVAT could be recovered by activating AMPK (Zaborska et al., 2017). However, to date, few studies have addressed how HFD modulates the anticontractile activity of PVAT independent of the endothelium and the role of AMPK in this effect. We hypothesized that AMPK expressed in PVAT would act as a protective mechanism to reduce some of the deleterious effects of high fat diet (HFD) on vascular function.

## Methods

### Animal model

Mice used in this study were housed in single-sex groups of 5–6 mice per cage at the Central Research Facility at the University of Glasgow and kept on 12 h cycles of light and dark and at ambient temperature. Wild type (Sv129-WT) mice were purchased from Harlan Laboratories (Oxon, UK). AMPKα1 KO mice were kindly supplied by Benoit Viollet (Institut Cochin, Paris, France) and the generation of these animals has been described before (Jørgensen et al., 2004). All animal experiments were performed in accordance with the United Kingdom Home Office Legislation under the Animals (Scientific Procedure) Act 1986 (project licenses 60/4114 and 70/8572 which were approved by the Glasgow University Animal Welfare and Ethical Review Board) and guidelines from Directive 2010/63/EU of the European Parliament on the protection of animals used for scientific purposes.

In all experiments, age-matched male and female WT and KO mice were used as preliminary experiments demonstrated no gender difference in aortic responses to contractile and relaxant agents (data not shown). WT and KO mice were randomly divided into two groups and fed either a normal diet [ND; *n* = 14(WT) and *n* = 14(KO)] or a high fat diet [HFD; *n* = 15(WT) and *n* = 15(KO)] for 12 weeks starting at 8 weeks of age. The high-fat diet (Western RD) was purchased from SDS (SDS diets, U.K) and contained: fat 21.4%, protein 17.5% and carbohydrate 50% (42% kcal fat). Body weight was monitored weekly and blood pressure (BP) was measured every 4 weeks using tail cuff plethysmography (Visitech systems, North Carolina, USA). Food intake was assessed by weighing the remaining food in the hopper of each cage. At the end of 12 weeks mice were fasted for 16 h before a glucose tolerance test (Mancini et al., 2017) and were then euthanised by a rising concentration of CO_2_. Blood was obtained by cardiac puncture and blood glucose measured using a portable glucose monitoring system (Ascensia CONTOUR blood glucose monitoring system, Bayer HealthCare). Serum insulin concentration was determined using a rat/mouse insulin ELISA kit (Millipore) according to the manufacturer's instructions in which the absorbance at 485 nm was determined using a FLUOstar OPTIMA microplate reader (BMG Labtech, Germany). Mean absorbance was determined from duplicate samples and concentration calculated by comparison to the standard curve.

For experiments involving the analysis of tissues, the thoracic aorta and spleen (used as a positive control) were removed and placed in ice-cold oxygenated (95% O_2_:5% CO_2_) Krebs' solution of the following composition: 118 mM NaCl, 4.7 mM KCl, 1.2 mM MgSO_4_, 25 mM NaHCO_3_, 1.03 mM KH_2_PO_4_, 11 mM glucose, and 2.5 mM CaCl_2_.

### Histological analysis

To determine the effect of HFD on PVAT morphology, freshly isolated thoracic aortae with intact PVAT from WT and KO mice were placed in 10% zinc formalin and fixed overnight. Arteries were processed through a gradient of alcohols to Histoclear and embedded vertically in paraffin wax before being sectioned on a microtome at 5 μm. Haematoxylin and eosin staining was performed and sections visualized by light microscopy.

Immunohistochemistry using rat anti-MAC2 (#CL8942AP, Cedarlane, UK), rabbit anti-AMPKα (#ab131512, Abcam) and anti-phospho-AMPKα Thr172 (#2535, Cell Signaling Technology) antibodies was utilized to detect the presence of inflammatory cells as well as the effect of HFD on AMPK phosphorylation. In brief, aortic rings and spleens from WT and KO mice fed ND or HFD were fixed overnight in 10% acetic zinc formalin. Sections (5 μm) on slides were deparaffinised and endogenous peroxidase activity blocked by immersing in 3% (v/v) H_2_O_2_ in methanol for 20 min. Non-specific antibody binding was blocked using 10% non-immune goat serum (Histostain Plus bulk kit blocking solution, Invitrogen) or normal rabbit serum (Vector labs; MAC2 antibody) for 1 h at room temperature and primary antibodies were then added overnight at 4°C. Antibodies were diluted in 1% (w/v) BSA in PBS and used at a concentration of 1:5000 (MAC2) or 1:100 (phospho-AMPKα). Secondary antibody (rabbit anti rat, Vector Labs, UK or biotinylated anti-rabbit, Histostain bulk kit) was incubated for 1 h at room temperature and antibody binding was visualized using DAB (3,3′ diaminobenzidine) chromogenic substrate (Vector Laboratories) and haematoxylin counter stain. Sections were photographed using AxioVision microscope software (Zeiss, Germany).

### Real-time PCR

Expression of M1 and M2 macrophage markers and adiponectin mRNAs in the PVAT was evaluated using real-time PCR as described elsewhere (Skiba et al., 2017). Briefly, total RNA was obtained from PVAT samples using RNeasy Lipid Tissue Mini Kit (Qiagen) and measured by Nanodrop 2000 (Thermo Fisher Scientific). Reverse transcription of 1 μg RNA was performed using High Capacity cDNA Reverse Transcription Kit (Applied Biosystems). mRNA expression of chosen genes in PVAT was analyzed using TaqMan® probes and TaqMan® Real-Time PCR Master Mix (Thermo Fisher Scientific). Expression of mRNA for *Tnf-*α and housekeeping gene *Gapdh* were analyzed using Fast SYBR® Green Master Mix (Thermo Fisher Scientific) and primers (Eurofins) shown in Supplementary Table 1. Reactions were prepared and run on 384-well plates on the QuantStudio™ 7 Flex Real-Time PCR System using a standard protocol and mRNA expression was analyzed using QuantStudio™ Real-Time PCR Software. All data were normalized to levels of *Gapdh* mRNA and relative quantification was calculated as 2^−ΔCt^. Details of the probes and primers used are listed in Supplementary Table 1.

### Protein expression/immunoblotting

Samples of thoracic aortic PVAT were dissected, weighed and lysed as previously described (Almabrouk et al., 2017). The protein content of WT and KO PVAT lysates derived from mice fed either ND or HFD was calculated by Coomassie Plus Protein Assay Reagent (Perbio, USA) against a BSA standard curve. Protein samples were resolved by SDS-PAGE, transferred to nitrocellulose membranes and incubated overnight at 4°C with mouse anti-GAPDH (Ambion AM4300) antibodies, or rabbit anti-AMPKα (Cell Signaling Technology #2603), anti-phospho-AMPK Thr172 (Cell Signaling Technology #2535), anti-acetyl CoA carboxylase (ACC) (Cell Signaling Technology #3676) and anti-phospho-ACC Ser79 (Cell Signaling Technology #3661) antibodies. All primary antibodies were diluted 1:1000 in 50% (v/v) TBS, 50% (v/v) Odyssey®-Block (LI-COR, USA). Immunolabelled proteins were visualized using infrared dye-labeled secondary antibodies and an Odyssey Sa Infrared Imaging System (LI-COR, USA).

### Adiponectin elisa

To examine the effect of HFD on adiponectin release from PVAT, conditioned media samples from WT and KO PVAT from ND and HFD groups were prepared according to the method of Almabrouk et al. (2017). Briefly, PVATs (20 mg) were isolated, weighed and incubated in 1 ml of aerating Krebs' solution at 37°C. Adiponectin content of the conditioned media were analyzed by adiponectin/Acrp30 Quantikine ELISA Kit (MRP300, R&D systems, Abingdon, Oxfordshire). Adiponectin was detected as a colourimetric reaction product by measuring absorbance of the ELISA plate at 450 nm with wavelength correction using a FLUOstar OPTIMA microplate reader (BMG Labtech, Germany). The mean absorbances for the samples were measured in duplicate and the adiponectin concentration was determined by comparison with the standard curve.

### Small vessel wire myography

WT and KO thoracic aortae from ND and HFD groups were cut into 2 mm rings with some rings cleaned of PVAT, and others left with the PVAT intact. In all experiments, the endothelium was removed by gently rubbing the interior of the vessel with fine wire and its absence was confirmed by lack of vasodilation (<10%) in response to 10^−6^ M acetylcholine. Artery rings were mounted on two stainless steel pins in a four channel wire myograph (Danish Myo Technology). Vessels were incubated in Krebs' at 37°C and gassed continuously with 95% O_2_ and 5% CO_2_. Rings were set at a pre-determined optimum resting force of 9.8 mN (Weingärtner et al., 2015) and allowed to equilibrate for 30 min prior to use. After calibration, arterial rings were challenged by addition of 40 mM KCl to sensitize the vessels and then contracted using 30 nM 9,11-dideoxy-9α,11α- methanoepoxy PGF2α (U46619, Tocris) before commencing experiments. Cumulative concentration–response curves to the K^+^ channel opener cromakalim (1 × 10^−9^ to 1 × 10^−6^ M; Sigma-Aldrich, Poole, UK), added at 10 min intervals were constructed. Data were expressed as a percentage loss of U46619-induced tone.

### Mouse carotid artery injury

To further investigate the role of AMPK in the PVAT on vessel inflammation, we used the mouse carotid wire injury model; characterized by endothelial denudation and infiltration of inflammatory cells throughout the vessel wall (Tennant et al., 2008; Greig et al., 2015). Briefly, mice (*n* = 5 WT and *n* = 4 KO fed on normal chow) were anesthetized and maintained on 1% isoflurane throughout. The left carotid artery was surgically exposed and injured luminally in WT and AMPKα1 KO mice using a flexible nylon wire. Animals were recovered with suitable analgesic cover and kept for 7 days. The right carotid artery served as a non-injured contralateral control. Arteries were removed, fixed, and processed for histological analysis and ICC as already outlined.

### Statistical analysis

All results are expressed as mean ± standard error of the mean (SEM) where n represents the number of experiments performed or number of mice used. Data were analyzed with GraphPad Prism 5.0 software. When comparing two or more variables two-way ANOVA (analysis of variance) tests were used. When comparing two or more data groups (contraction data), two-way ANOVA followed by Newman–Keuls post hoc test was used. In all cases, a *p*-value of < 0.05 was considered statistically significant.

## Results

### Effect of HFD on weight, food intake, and blood pressure

At the end of the 12 week period of feeding there was a significant increase in body weight in WT mice on HFD compared to mice fed ND (Figure S1A). Fasting blood glucose and plasma insulin measured at the end of the study period both showed a tendency toward an increase in the WT HFD group, as did incremental area under the curve (AUC) following the glucose tolerance test (Table 1 and Figures S1C,D). In the KO mice, weight gain following HFD was similar to that observed in WT mice (Figure S1A). Similarly, fasting blood glucose, plasma insulin (Table 1) and incremental AUC following a GTT were no different from the values seen in WT mice fed either diet. Systolic blood pressure was unchanged in any group over the course of the 12 week study period (Figure S1B).

**Table 1:** Effect of 12 weeks of high fat diet (HFD) on plasma glucose, insulin and AUC following a glucose tolerance test.

**Parameter**	**WT ND (*n* = 10)**	**WT HFD (*n* = 11)**	**KO ND (*n* = 10)**	**KO HFD (*n* = 11)**
Blood glucose (mmol/l)	3.8 ± 0.3	4.7 ± 0.6	4.1 ± 0.3	4.9 ± 0.6
Insulin (ng/ml)	0.6 ± 0.04	0.9 ± 0.1	0.6 ± 0.03	0.7 ± 0.03
GTT (AUC)	1035 ± 91.66	1187 ± 141.4	1046 ± 97.88	1220 ± 131.3

### PVAT morphology and inflammation

**Figure 1:**
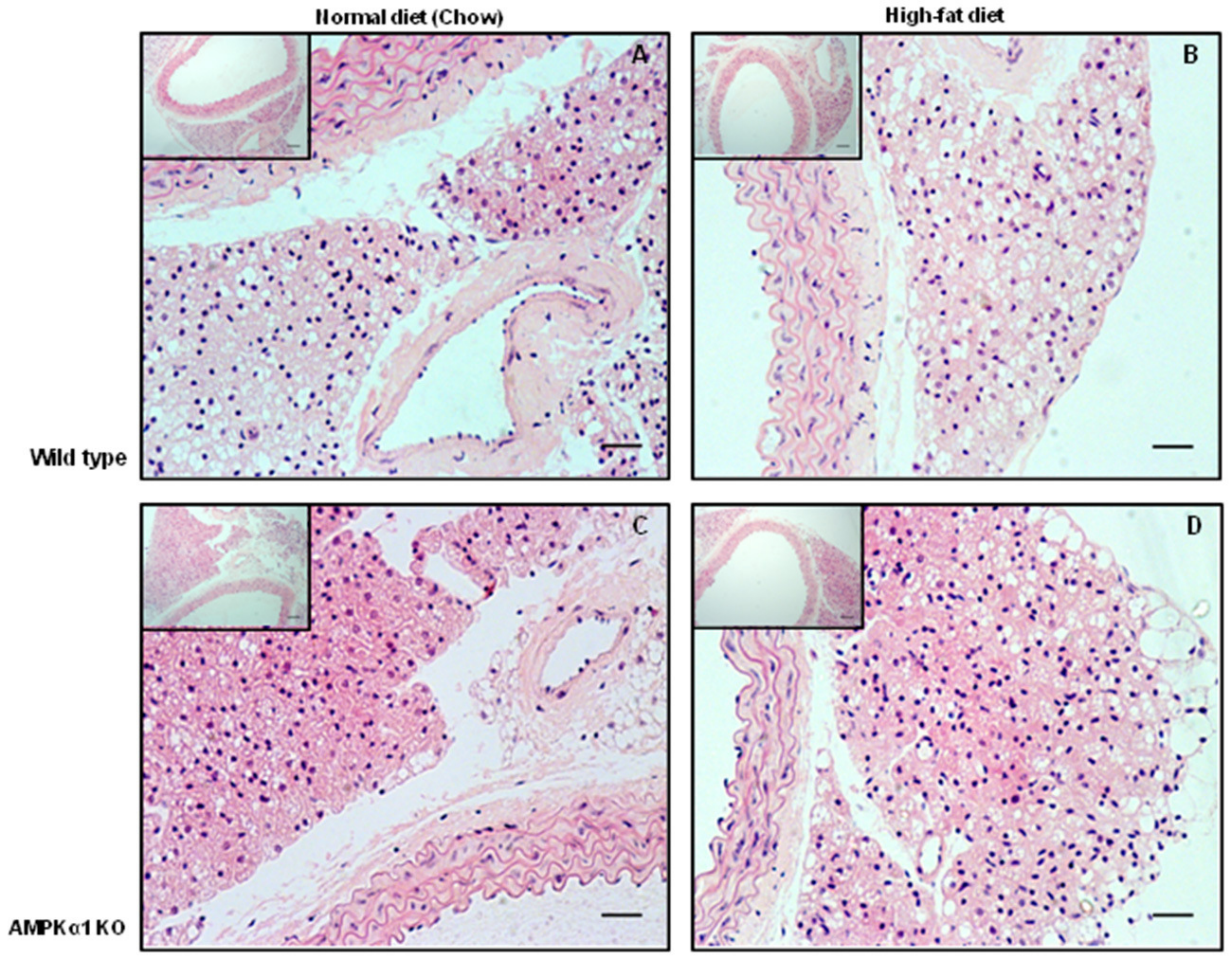
Effect of 12 weeks high-fat diet on thoracic aorta PVAT. Aortic rings from wild type mice **(A,B)** and AMPKα1 knockout mice **(C,D)** were harvested from mice fed normal diet (chow) or high fat diet and stained with H&E. Nuclei appear blue/purple whereas cytoplasm is stained pink. Scale bar = 20 μm. Representative images of *n* = 5 separate animals per group.

**Figure 2:**
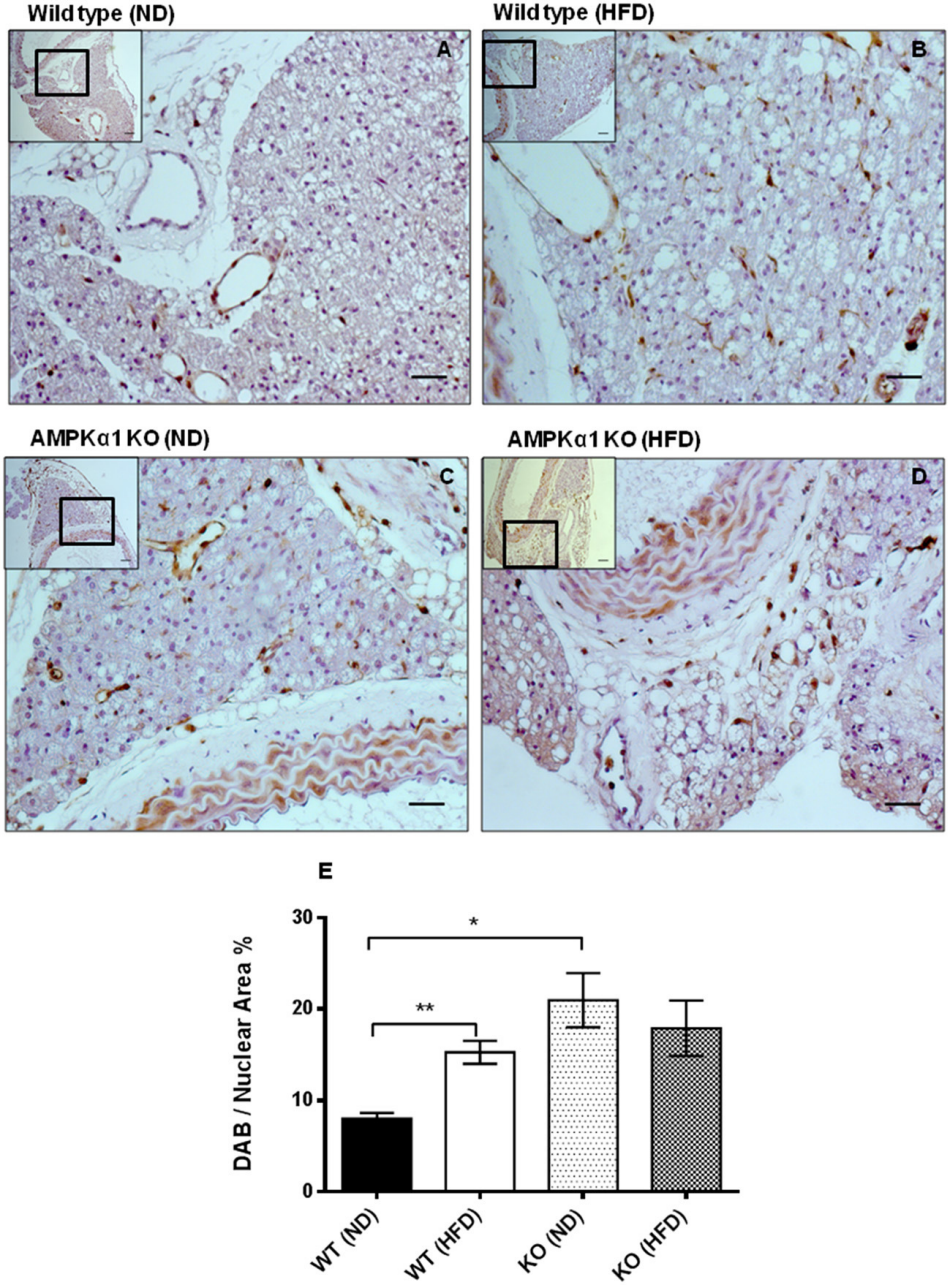
Effect of high-fat diet on macrophage marker (MAC2) expression in thoracic aortic PVAT. Aortic rings from wild type mice **(A,B)** and AMPKα1 knockout mice **(C,D)** were harvested from mice fed normal diet (ND) **(A,C)** or high fat diet (HFD) **(B,D)** and immunostained with anti-MAC2 antibody with haematoxylin counterstain. Scale bar = 20μm. Images shown are representative of at least *n* = 5 separate animals per group. **(E)** Histogram showing quantitation of immunostaining data. Data were expressed as percentage of stained cells to total nuclear area in the section. ^**^p <0.01 vs WT (ND); ^*^p < 0.05 vs WT (ND).

**Figure 3:**
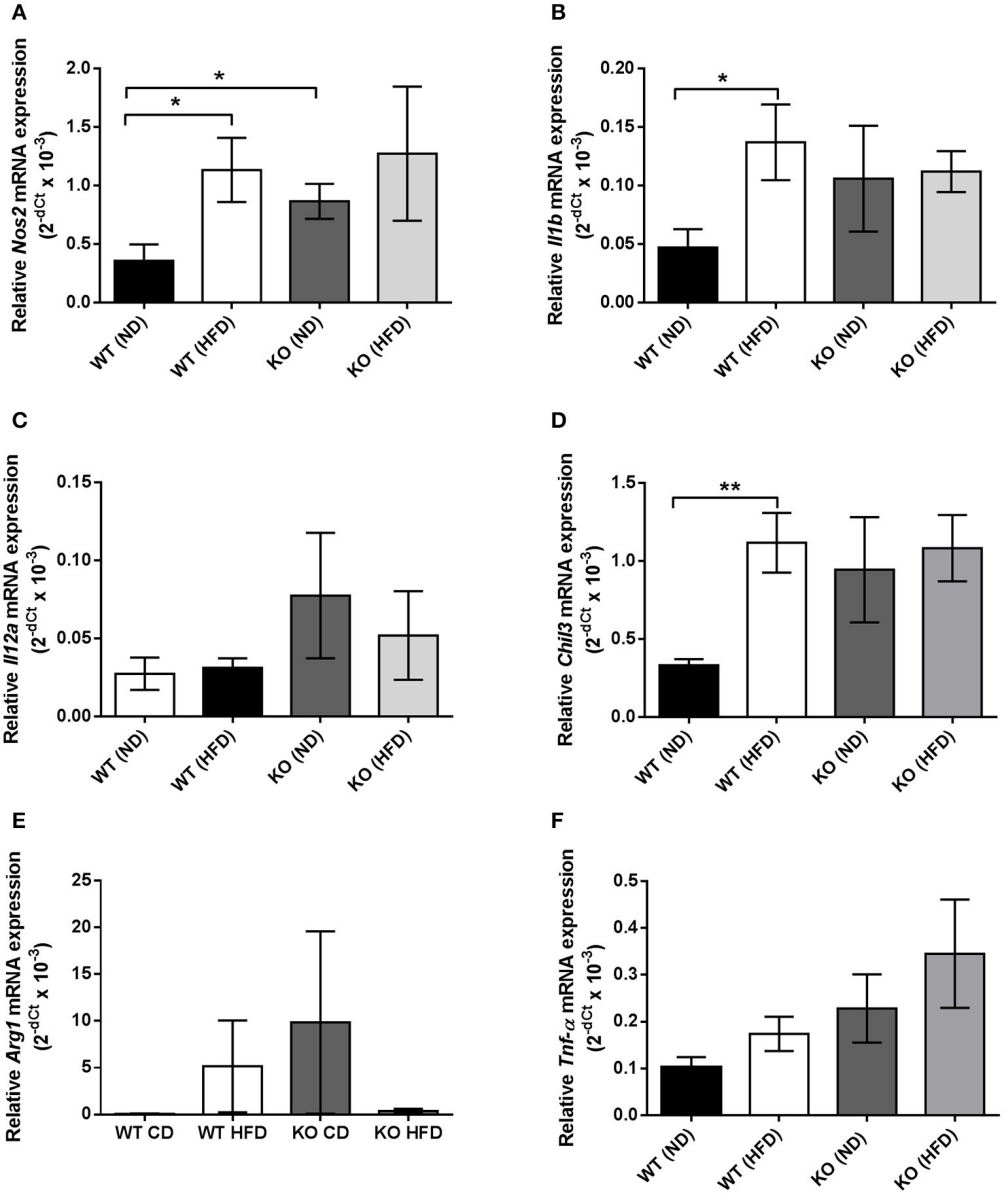
Quantification of macrophage marker expression within thoracic PVAT samples using RT-PCR. **(A,B)**, HFD significantly raised mRNA expression of M1 markers *Nos2* and *Il1b*, encoding iNOS and IL-1β in WT PVAT but had no effect in PVAT from AMPK α1 KO mice. KO PVAT had a higher expression of iNOS compared to WT PVAT in mice fed ND. **(C)** Expression of *Il12a*, encoding IL-12 was unchanged following HFD in either WT or KO mice. **(D)** HFD also significantly increased expression of the M2 marker *Chil3*, encoding YM1 in WT but not KO mice. **(E)** Expression of the M2 marker *Arg1*, encoding arginase was unchanged following HFD in either WT or KO mice. Similarly, expression of *Tnf-*α, encoding TNF-α was unchanged following HFD in either WT or KO mice **(F)**. Values are expressed as means ± SEM, *n* = 4 for all groups; ^*^p < 0.05 and ^**^p < 0.01 vs. WT (ND). iNOS- inducible nitric oxide synthase; IL-1β- interleukin 1β; YM1, Beta-N-acetylhexosaminidase.

In haematoxylin and eosin stained samples of thoracic aortic PVAT from both WT and KO mice, the PVAT was composed of adipocytes with the morphological features of brown adipose tissue (BAT). After 12 weeks of HFD, the PVAT did not appear grossly altered although there were some cells containing larger lipid droplets with the appearance of white adipocytes (Figure 1). Immunohistochemistry for the macrophage marker MAC2 was used to identify the effect of HFD on monocyte/macrophage numbers within the PVAT. In WT mice, there was very little positive MAC2 staining within the PVAT after 12 weeks of ND but this was significantly increased in mice fed HFD (Figure 2). In KO mice fed ND, there was significantly more positive MAC2 staining compared to WT mice on ND, indicating that lack of AMPKα1 increases monocyte/macrophage numbers within PVAT and that AMPK may protect against deleterious changes in immune cell infiltration in PVAT. In KO mice fed HFD, there was no further increase in MAC2 staining (Figure 2). To study macrophage phenotype in more detail, real-time PCR was used to identify mRNA levels of markers of M1 and M2 macrophages in homogenized samples of PVAT. In WT mice fed HFD there was an increased expression of some M1 macrophage marker mRNAs (*Nos2* and *Il1b*; Figures 3A,B) while others (*Il12a*) remained unchanged (Figure 3C). Interestingly, KO mice fed ND exhibited increased *Nos2* when compared to WT mice fed ND. HFD did not cause any further increase in these markers in KO mice (Figures 3A,B). Within the M2 markers, levels of *Chil3* mRNA were increased in WT mice after 12 weeks of HFD (Figure 3D), while *Arg1* mRNA remained unchanged (Figure 3E). Levels of *Tnf-*α were not changed in KO mice compared to WT mice and HFD had no significant effect (Figure 3F).

### AMPK levels and phosphorylation in PVAT

**Figure 4:**
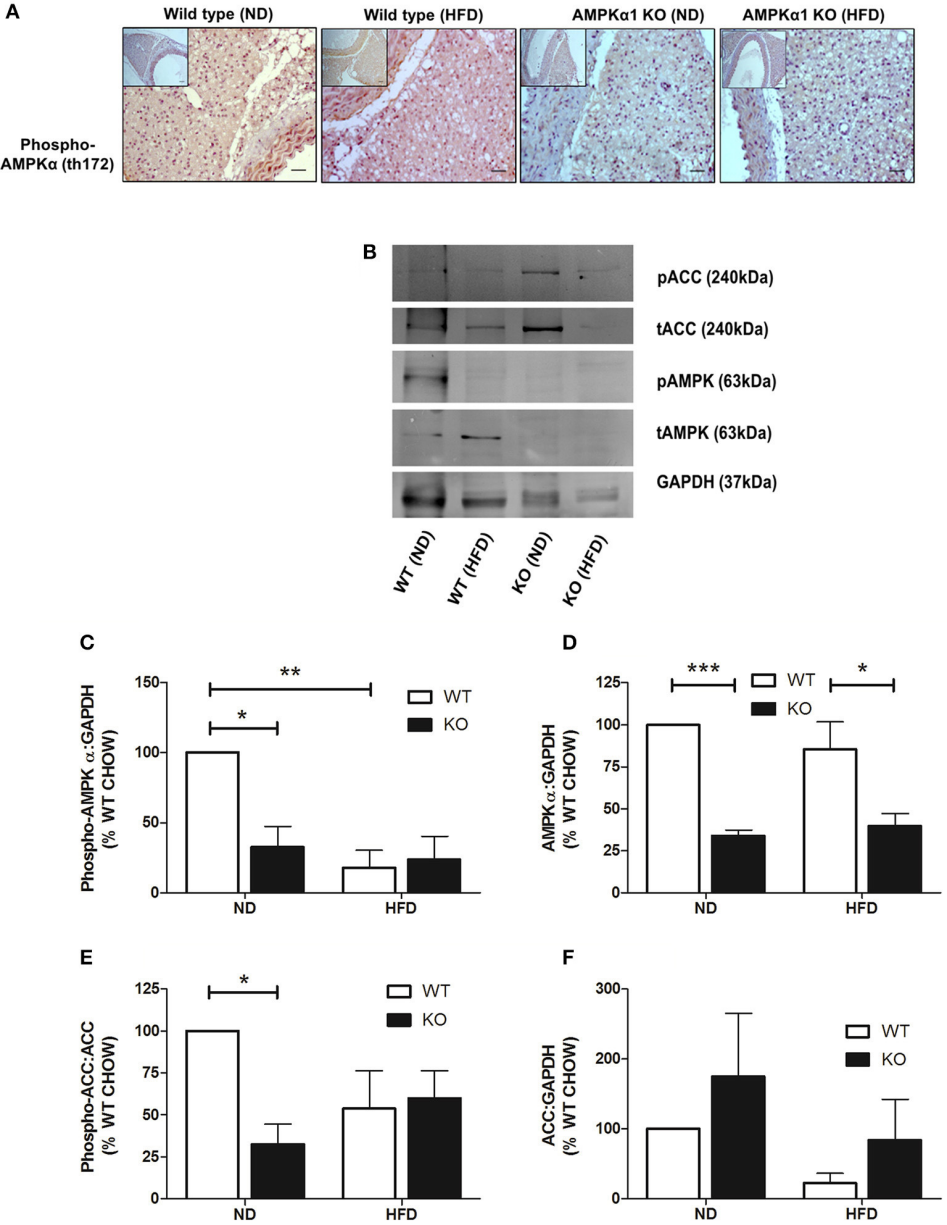
Effect of HFD on levels and phosphorylation of AMPK in PVAT. Sections of thoracic aorta from WT and KO mice on normal or high-fat diet were probed using anti-phospho-AMPKα Thr172 antibodies **(A)**. There was significantly less staining in KO tissue compared to WT but feeding high-fat diet had no obvious effect on phospho-AMPKα levels. Scale bar = 20μm, representative images of at least *n* = 3 separate mice per group. **(C–F)** lysates of PVAT from WT and KO mice fed ND and HFD were immunoblotted with the indicated antibodies. **(B)** Representative immunoblots. **(C–F)** Quantitative analysis of immunoblots, expressed as the ratio of phosphorylated AMPKα **(C)**, AMPKα **(D)**, and total ACC **(F)** relative to GAPDH. **(E)** Quantitative analysis of phosphorylation of the AMPK substrate, ACC, relative to total ACC levels. ^*^p < 0.05, ^**^p < 0.01or ^***^p < 0.001 vs. WT (ND), *n* = 3 for all data sets.

In WT mice, staining for phospho-AMPKα Thr172 was found throughout the PVAT surrounding the aorta. HFD did not appear to alter staining intensity noticeably. In the KO mice, staining intensity for phospho-AMPKα Thr172 was markedly lower compared to the WT and HFD did not change the apparent intensity of the staining (Figure 4A). Immunoblotting was used to quantify the levels of phosphorylated AMPKα and the AMPK substrate, ACC in homogenized PVAT samples (Figure 4B). Compared to WT mice fed ND, mice fed HFD exhibited reduced levels of phosphorylated AMPKα without altering total levels of AMPKα relative to GAPDH (Figures 4C,D). Furthermore, PVAT homogenates from WT mice fed HFD exhibited a tendency toward reduced phosphorylated ACC relative to total ACC compared with WT mice fed ND, yet, this did not achieve statistical significance (Figure 4E). As expected, KO mice on either diet exhibited markedly reduced levels of AMPKα and phosphorylated AMPKα (Figures 4C,D). Furthermore, this was associated with reduced AMPK activity as assessed by ACC phosphorylation relative to total ACC (Figure 4E), without altering total ACC levels (Figure 4F).

### Adiponectin production by PVAT

**Figure 5:**
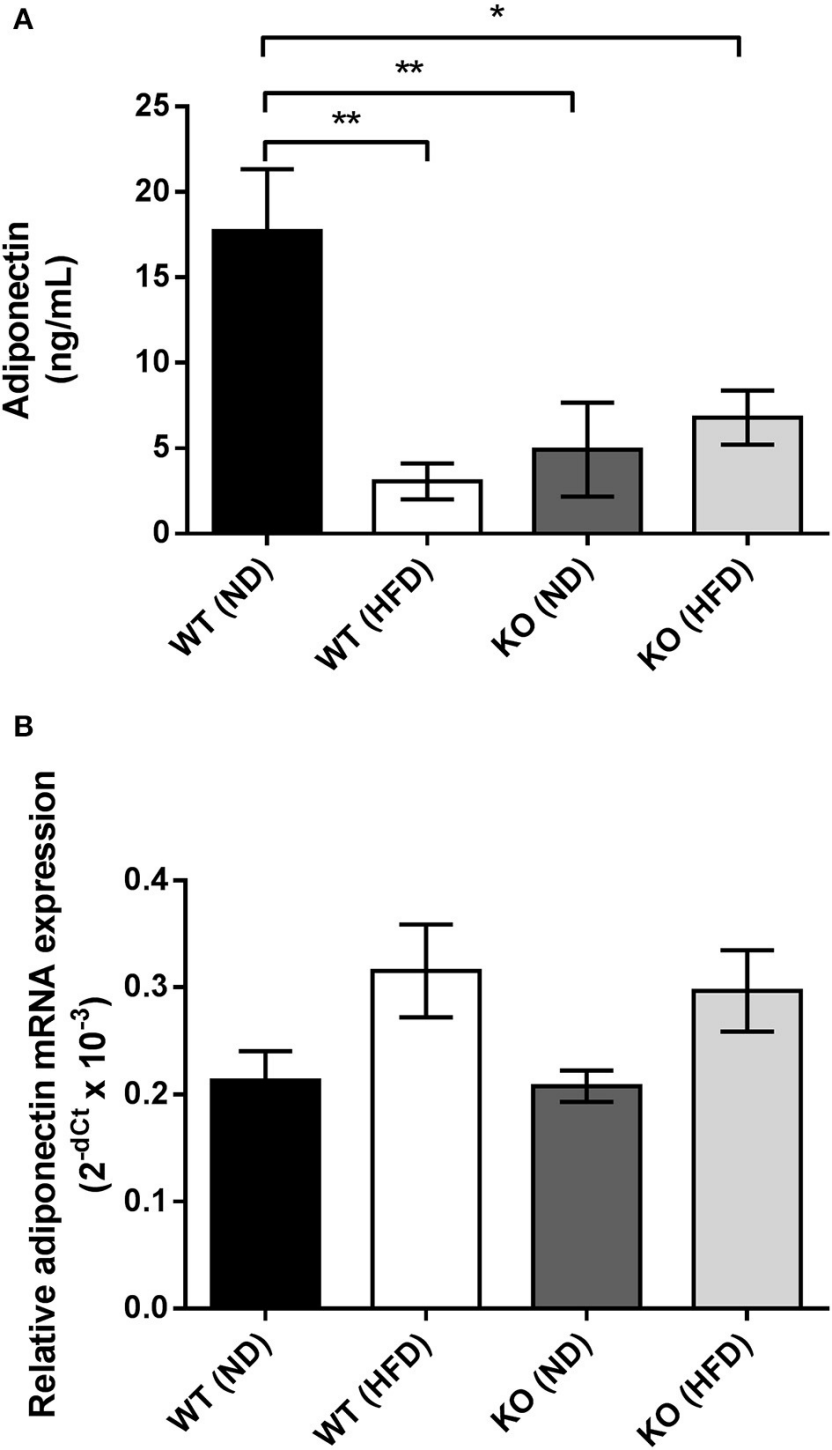
Effect of HFD on adiponectin secretion and mRNA expression in PVAT. **(A)** Conditioned medium was collected from WT and KO PVAT from mice fed ND or HFD and adiponectin levels assessed by ELISA (*n* = 6 for all groups). ^*^p < 0.05 and ^**^p < 0.01 vs. WT ND. **(B)** Adiponectin mRNA expression was assessed by qPCR in samples of aortic PVAT from WT and KO fed either ND or HFD (*n* = 4 for all groups). No significant changes in adiponectin gene expression were detected.

HFD caused a significant reduction (~70%) in the adiponectin content of conditioned media (CM) collected from PVAT of WT mice compared to mice fed ND (Figure 5A). CM from KO mice fed ND had significantly lower adiponectin content compared to WT mice, yet HFD caused no further alteration of adiponectin levels in CM from PVAT of KO mice (Figure 5A). To further investigate the changes in adiponectin caused by HFD, RT-PCR was used to quantify adiponectin mRNA expression. In WT mice, feeding HFD for 12 weeks had no effect on adiponectin mRNA expression (Figure 5B) and in KO mice, expression was not significantly different compared to WT mice and feeding HFD had no effect. This suggests that HFD or AMPKα1 knockout significantly attenuates adiponectin secretion by the PVAT without affecting gene expression.

### Anticontractile effect of PVAT and importance of AMPK

**Figure 6:**
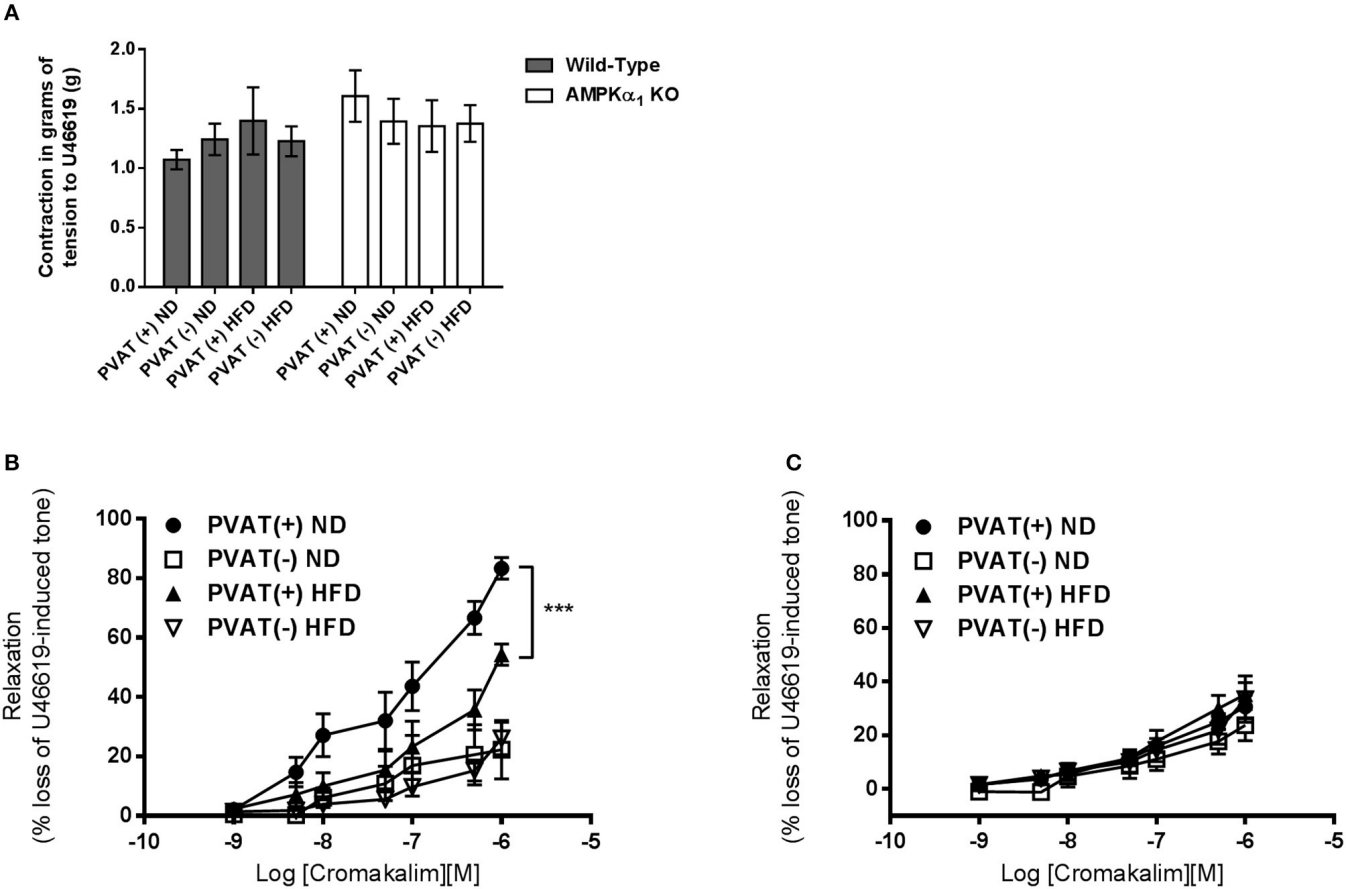
Effect of HFD on the anticontractile effect of aortic PVAT. **(A)** There was no significant difference in contractile force in response to 30nM U46619 in rings from WT or KO mice and this was not influenced by 12 weeks of HFD or the presence or absence of intact PVAT on the artery ring. Dose response curves to cromakalim were constructed in preconstricted aortic rings from WT mice **(B)** or AMPKα1 knockout mice **(C)**. All rings were denuded of endothelium and either had PVAT removed (–) or left intact (+). Intact PVAT had an anticontractile effect in WT mice which was attenuated by high fat diet feeding. HFD had no effect on relaxation in rings without attached PVAT **(A)**. In KO mice, the PVAT did not have an anticontractile effect and HFD did not alter this **(B)**. ^***^p < 0.001 vs. PVAT(–) (ND); *n* = 6–7.

The presence or absence of the vascular endothelium did not affect the contractile response to U46619 in WT or KO aortic rings (data not shown). In endothelium-denuded vessels, the contraction of WT aorta without PVAT to 30 nM U46619 was 1.2 ± 0.3 g (*n* = 7) and this was not significantly affected by 12 weeks of HFD (Figure 6A). Similarly, in WT vessels with intact PVAT, contraction to 30 nM U46619 was unaffected by HFD (1.4 ± 0.3 g; *n* = 6 vs. 1.1 ± 0.1 g; Figure 6A). KO mice showed a similar contraction in response to U46619 and this was unaffected following HFD in rings either with or without intact PVAT (Figure 6C).

In WT mice, the maximum relaxation to cromakalim was significantly increased in aortic rings with intact PVAT (83.3 ± 3.6%, *n* = 7 vs. 27.6 ± 2.8%, *n* = 7; *p* < 0.05). After 12 weeks of HFD, the maximal relaxation to cromakalim in aortic rings with intact PVAT was significantly attenuated (around 30%) compared to mice fed ND (Figure 6B) while in aortic rings without PVAT, the diet had no effect on relaxation, suggesting it is dysfunction of the PVAT caused by HFD which attenuates the anticontractile effect. In the AMPKα1 KO mice fed normal diet, the anticontractile effect of the PVAT was absent and maximal responses to cromakalim were not significantly different between vessels with or without intact PVAT. In KO mice fed HFD, there was no significant change in the relaxation to cromakalim (Figure 6C).

### Carotid injury—effect on inflammation in PVAT

**Figure 7:**
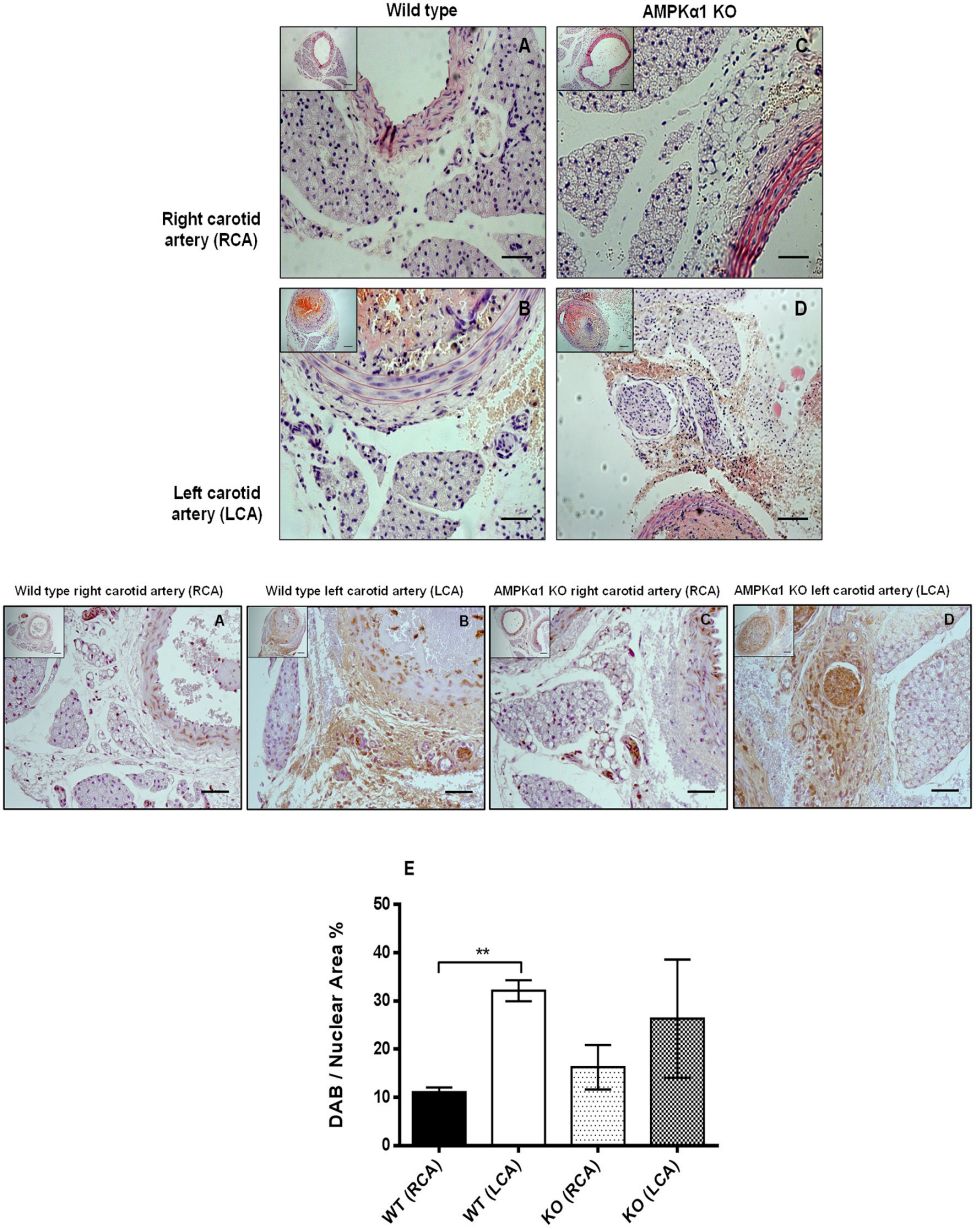
Effect of wire injury on carotid PVAT morphology and inflammation in WT and AMPKα1 KO mice. **(A–D)** haematoxylin and eosin stained sections harvested from WT and KO right carotid arteries (non-injured) and left carotid arteries (injured). Representative images from *n* = 4–5 separate animals are shown. There were no obvious differences in PVAT morphology caused by wire injury in either the WT or KO mice. Scale bar; 20 μm. Middle panel- representative WT and KO right and left carotid arteries with intact PVAT stained with anti- MAC2 antibody and counterstained with haematoxylin. Wire injury caused a dramatic increase in MAC2 expression in the PVAT of WT carotid arteries. In KO mice, there was a trend toward increased MAC2 expression in the non-injured vessels and also in the injured vessels compared to WT mice but this failed to reach significance. **(E)** Histogram showing quantification of MAC2 staining calculated as percentage of stained cells to total nuclear area in the section. ^**^p < 0.001 vs. WT RCA.

We next sought to investigate if vascular injury exerts effects on PVAT and if this is mediated by AMPK. Using the carotid artery wire injury model we examined whether PVAT lacking AMPKα1 responded differently to vascular injury and associated vessel inflammation. PVAT surrounding the carotid artery appeared very similar to that surrounding the thoracic aorta. Haematoxylin and eosin staining revealed that WT PVAT consisted of adipocytes with the characteristic multiple lipid vacuoles and central nuclei which is characteristic of brown adipocytes and this was similar in the wire injured left carotid samples (Figures 7A,B). In KO mice, the PVAT had a similar BAT-like appearance and this was unchanged 7 days after wire injury (Figures 7C,D). Immunohistochemistry using the antibody against MAC2 showed significant macrophage infiltration in WT injured carotid arteries in comparison with the control right carotid (Figure 7E). In KO mice, there was a trend toward greater MAC2 expression in the PVAT of the non-injured vessels but there was no significant increase caused by wire injury (Figure 7E).

## Discussion

This study investigated the effect of HFD on PVAT regulation of conduit artery tone in WT and AMPKα1 KO mice. One of the novel findings of this study was that the anticontractile effect of PVAT in aortic rings with no endothelium was significantly diminished in WT mice fed a HFD compared to those maintained on chow diet. The loss of anti-contractile function could be due in part to a reduction in PVAT-derived adiponectin release caused by AMPK dysfunction and/or inflammation within the PVAT. This is supported by data obtained in the AMPKα1 KO mouse which displayed dysfunctional aortic PVAT with increased macrophage infiltration and a lack of anticontractile activity even in the absence of HFD.

Both WT and KO animals fed the HFD gained weight to a greater extent than those fed normal diet; indicative of the obesogenic nature of the diet. However, other cardiometabolic parameters such as plasma insulin and glucose as well as systolic arterial blood pressure were not significantly elevated in either of the HFD groups compared to groups fed the normal diet. This is in contrast with a previous study in mice on a 54% kcal fat diet for only 8 weeks (da Silva Franco et al., 2017). In terms of plasma glucose and insulin, most studies report a rise induced by HFD, even after only 4 weeks of feeding (Guilford et al., 2017), but others have found no change after 6 weeks (60% kcal of fat diet; Zhang et al., 2017). Our study used a diet with a lower percentage of kcal as fat (42%) and we cannot rule out changes occurring if feeding duration was increased beyond 12 weeks as both plasma insulin and glucose showed trends toward an increase in both HFD groups. However, it is clear that knockout of the AMPKα1 gene did not affect the cardiometabolic paramaters or the morphological appearance of the PVAT.

The mechanisms underlying the effect of AMPK in regulating adipose tissue mass are poorly characterized. Adipose tissue mass expansion occurs as a consequence of either an increase in adipocyte number as a result of enhanced adipogenesis, an increase in cell size due to fat deposition in pre-existing cells, or a combination of both. It has been reported that the increase in adipose tissue mass in AMPKα2 knockout mice was due to an increased triglyceride accumulation in the pre-existing adipocytes rather than an increase in cell number or differentiation as no changes in the expression of adipocyte transcription factors, PPARγ, C/EBPα, or the mature adipocyte markers, including aFABP/aP2, were reported (Villena et al., 2004). The model used in the current study is a global AMPKα1 knockout mouse and there was no evidence of increased adipose tissue mass and KO animals on the HFD gained weight at the same rate as the WT mice. Weight gain and development of obesity is dependent on energy balance.

The main focus of this study was the effect of HFD on aortic PVAT and here, HFD caused a marked increase in the number of macrophages in WT mice as detected by the MAC2 marker (Figure 2). The gross morphological appearance of the PVAT was not different between WT and KO groups but 12 weeks of HFD did cause the appearance of some adipocytes with larger lipid droplets in the PVAT (Figure 1) and this is likely related to an overall increase in the adiposity of the fat-fed animals as evidenced by the weight gain. RT-PCR was used to differentiate between M1 and M2 type macrophages within the PVAT. In the WT group, the HFD increased the expression of M1 markers (iNOS and IL-1β) in comparison with ND fed mice. M1 macrophages have pro-inflammatory and anti-angiogenic properties and generation of inflammatory cytokines within the PVAT may play a role in the adipose tissue microenvironment and affect generation of adipokines such as adiponectin. Indeed a recent study also found increases in the PVAT content of M1 macrophages in the ApoE^−/−^ mouse model of atherosclerosis, suggesting that macrophage polarization in the artery wall can drive vascular disease (Skiba et al., 2017). In the KO mouse there was a significantly increased expression of iNOS but HFD did not cause any further increase in the these markers and this may be indicative of the already inflamed PVAT in the KO or that AMPKα1 is necessary for macrophage polarization. In support of this, rats with chronic kidney disease have reduced AMPK activity in macrophages which disturbs macrophage polarization and this can be restored by activating AMPK (Li et al., 2015). Another possibility is that adipokines produced by the PVAT can affect macrophage polarization. Indeed, human adipocyte-conditioned media was found to modulate the expression profile of macrophages via AMPK activation and expression of angiotensin-converting enzyme (ACE) (Kohlstedt et al., 2011). However, it should be noted that one M2 marker was also raised in WT mice fed HFD which could suggest recruitment of both M1 and M2 macrophages rather than unequivocal polarization toward an M1 phenotype. This may also be supported by the fact that the expression data are not normalized to a general macrophage marker, thus the increased expression of some of these markers could simply be due to the larger number of macrophages present in the WT PVAT following HFD and further work is required to study this in more detail.

To investigate this further, we measured adiponectin production in adipocyte-conditioned media from aortic PVAT and also studied AMPK levels in homogenized PVAT samples to assess if this was affected by the HFD and if it could underlie the inflammatory changes. Previous studies have shown that even very short periods of HFD cause a rise in the inflammatory adipocytokine leptin and chemokine MIP1α concomitant with a decrease in the expression of adiponectin, PPARγ, and FABP4 (Chatterjee et al., 2009). The data presented here also show a clear and significant reduction in adiponectin secretion caused by HFD in the WT mouse (Figure 5A) but with no significant change in adiponectin gene expression (Figure 5B), suggesting a deficiency in gene translation or adiponectin secretion caused by HFD. In agreement with our results Ketonen et al. also found no change in adiponectin mRNA expression in thoracic PVAT even after 8 months of 60% kcal fat diet in C57BL6/J mice (Ketonen et al., 2010).

Several lines of evidence report that AMPK may regulate adiponectin secretion by the PVAT via suppression of inflammatory cytokines such as TNF-α and IL-6 (Lihn et al., 2004; Tsuchida et al., 2005; Sell et al., 2006). Activation of AMPK with AICAR in human adipose tissue was associated with degradation of TNF-α and increased adiponectin gene expression (Lihn et al., 2004) while TNF-α and IL-6 are known to have inhibitory effects on adiponectin gene expression and release (Fasshauer et al., 2002, 2003). Moreover, TNF-α has been suggested to play a central role in regulating adiponectin levels (Greenberg et al., 1991) and suppressing TNF-α protein may be involved in the up-regulation of adiponectin mRNA levels (Lihn et al., 2004). Sell et al. reported activation of AMPK by AICAR and troglitazone was associated with reduction of IL-6, IL-8, MIP-1α/β, and MCP-1 and upregulation of adiponectin expression (Sell et al., 2006). Similar findings demonstrate that the expression of inflammatory genes including TNF-α, MCP-1, and macrophage antigen-1 in WAT was reduced in response to PPARα agonist rosiglitazone (Tsuchida et al., 2005). Thus, in the current study, the increase in the expression of macrophage markers and reduced adiponectin secretion reported in fat-fed WT and KO animals may be due to reduced AMPK activity leading to upregulation of inflammatory cytokines such as TNF-α and IL-6 and downregulation of adiponectin secretion by the PVAT. Indeed, in WT animals fed HFD, there was a significant reduction in activating Thr172 phosphorylation of AMPK, without any change in overall AMPK levels (Figure 4C). This indicates a reduction in AMPK activity as a result of feeding HFD and interestingly, this was phenocopied in animals lacking AMPKα1.

Functionally, we hypothesized that HFD may impair vessel relaxation. In a previous study we showed that PVAT exerts an anticontractile effect in murine aorta and that this is lost in AMPKα1 KO mice due to PVAT dysfunction with reduced adiponectin secretion in the KO mouse (Almabrouk et al., 2017). Indeed, in that study we found that the presence of PVAT also caused a slight reduction in contraction of WT but not KO aortic rings in response to the thromboxane agonist U46619. This was not replicated here and feeding either WT or KO mice HFD had no effect on contraction to U46619 (Figure 6A). However, in common with our previous study, presence of PVAT increased relaxation to cromakalim in WT but not KO aortic rings. Here we extend these findings to demonstrate that the anticontractile effects of PVAT can be attenuated in WT mice by 12 weeks of HFD (Figure 6). In vessels lacking PVAT, HFD had no significant effect on relaxation to the endothelium-independent vasodilator cromakalim and this was the case in both WT and KO mice. However, in vessels with intact PVAT, HFD attenuated relaxation in the WT group but not the KO group. This strongly suggests that the effect of HFD in WT mice was on the PVAT rather than on the medial VSMCs. Since the anticontractile effect was absent in KO mice, and unaffected by HFD, it also seems likely that AMPK within the PVAT is involved in mediating the anticontractile response, likely via generation of adiponectin which is a vasodilator in mouse aorta (Almabrouk et al., 2017). However, it must be noted that there are other targets of AMPK in addition to adiponectin and these cannot be ruled out from involvement in the loss of the anticontractile effect of PVAT in HFD-fed mice.

These results are consistent with previous studies reporting that PVAT-mediated anticontractile effect is impaired in HFD models (Nakagawa et al., 2002; Gao et al., 2005; Fesus et al., 2007; Greenstein et al., 2009; Marchesi et al., 2009; Ma et al., 2010; Payne et al., 2010; Owen et al., 2013). Gao et al. reported that the effect is lost in obese rats due to reduced release of relaxing factors despite the increased amount of PVAT around rat aorta (Gao et al., 2005). A loss of the anticontractile effect of PVAT was also reported in obese New Zealand mice (NZO), a model which is characterized by metabolic syndrome and larger amounts of PVAT. This was suggested to be due to changes in the expression of PVAT-derived factors other than adiponectin (Fesus et al., 2007). In the Ossabaw swine model of obesity there was an up-regulation of 186 PVAT-derived proteins associated with increased coronary contractility and these included transforming protein RhoA and calpastatin (Owen et al., 2013). HFD-induced obesity likely impairs PVAT-mediated anticontractile effects by promoting a marked proinflammatory shift in cytokines and chemokines associated with oxidative stress in the PVAT (Bailey-Downs et al., 2013). Although it wasn't studied here, PVAT inflammation and oxidative stress can also lead to endothelial dysfunction with decreased NO bioavailability and increased superoxide generation by uncoupled endothelial NO synthase in PVAT (Marchesi et al., 2009).

Since we found reduced AMPK activity in the PVAT of mice fed HFD it is tempting to speculate that AMPK acts as a protective mechanism against inflammation and loss of PVAT anticontractile function and that this effect is overcome in HFD-induced obesity. Indeed, AMPK activation inhibits multiple pro-inflammatory signaling pathways in cultured adipocytes (Mancini et al., 2017). Additionally, in KO mice, the PVAT already showed increased MAC2 expression following ND and HFD had no additional effect. These results further support the protective anti-inflammatory role of AMPK in PVAT. The absence of any difference between ND and HFD groups in the KO mice may be due to the fact that the PVAT of KO mice is already maximally infiltrated or perhaps as a result of a compensation mechanism by AMPKα2 complexes in the PVAT which prevent or modulate further inflammatory cell infiltration and this requires further investigation. Other stimuli which induce vascular inflammation also lack an effect in the AMPKα1 KO mouse and here we demonstrate that in the mouse carotid artery injury model. While injury to the WT carotid caused a strong infiltration of macrophages 7 days after injury, the KO carotid PVAT already had a trend toward increased macrophage infiltration and wire injury did not affect this; suggesting that the activity of AMPK in the PVAT prevents the PVAT becoming inflamed.

## Conclusion

In conclusion, HFD is associated with increased macrophage infiltration and polarization toward the M1 inflammatory phenotype, reduced AMPK activity and reduced adiponectin secretion by thoracic PVAT. These changes likely underlie the loss of the anti-contractile activity of the PVAT. Vascular AMPK, and in particular AMPK expressed in the PVAT may therefore protect the vessel against deleterious changes in response to HFD and may be a target to treat vessel inflammation seen in many cardiovascular diseases.

## Author contributions

TA, AW, AU, DS, HA, and OK: conducted the experiments, collected and analyzed the data and prepared the figures; TA: drafted sections of the paper; IS, RT, and SK: conceived and planned the experiments; SK: prepared the final version of the manuscript and SK, RT, TG, and IS: proof read the final version of the manuscript.

### Conflict of interest statement

The authors declare that the research was conducted in the absence of any commercial or financial relationships that could be construed as a potential conflict of interest.
