# Correction: High fat diet attenuates the anticontractile activity of aortic PVAT via a mechanism involving AMPK and reduced adiponectin secretion

**DOI:** 10.3389/fphys.2025.1750072

**Published:** 2025-12-19

**Authors:** Tarek A. M. Almabrouk, Anna D. White, Azizah B. Ugusman, Dominik S. Skiba, Omar J. Katwan, Husam Alganga, Tomasz J. Guzik, Rhian M. Touyz, Ian P. Salt, Simon Kennedy

**Affiliations:** 1 Institute of Cardiovascular and Medical Sciences, College of Medical, Veterinary and Life Sciences, University of Glasgow, Glasgow, United Kingdom; 2 Medical School, University of Zawia, Zawia, Libya; 3 Department of Physiology, National University of Malaysia Medical Centre, Kuala Lumpur, Malaysia; 4 Jagiellonian University College of Medicine, Krakow, Poland; 5 Department of Biochemistry, College of Medicine, University of Diyala, Baqubah, Iraq

**Keywords:** perivascular adipose tissue, AMPK, high-fat diet, adiponectin, anticontractile effect

An incorrect UK project licence number was included- 60/4224 should be 60/4114. A correction has been made to the section **Methods**, Animal Model, paragraph 1:

“All animal experiments were performed in accordance with the United Kingdom Home Office Legislation under the Animals (Scientific Procedure) Act 1986 (project licenses 60/4114 and 70/8572 which were approved by the Glasgow University Animal Welfare and Ethical Review Board) and guidelines from Directive 2010/63/EU of the European Parliament on the protection of animals used for scientific purposes”

The original article has been updated.

