# Supplementary material for: High Fat Diet Attenuates the Anticontractile Activity of Aortic PVAT via a Mechanism Involving AMPK and Reduced Adiponectin Secretion
Source: Front Physiol. 2018 Feb 9;9:51. doi: 10.3389/fphys.2018.00051 (PMC5812172; doi:10.3389/fphys.2018.00051)
Supplement: Supplementary file 1 [file Table1.DOCX]

Supplementary table 1- gene probes (upper table) and primers (lower table) used in RT-PCR experiments

| **Gene** | **TAQMAN probes (Catalogue number)** |
| --- | --- |
| Adiponectin (*Adipoq*) | Mm00456425_m1 |
| Ym1 (*Chil3*) | Mm00657889_mH |
| Arginase (*Arg1*) | Mm00475988_m1 |
| Interleukin 1β (*Il1b*) | Mm99999061_mH |
| Interleukin 12a (*Il12a*) | Mm00434169_m1 |
| iNOS (*Nos2*) | Mm00440502_m1 |
| GAPDH (*Gapdh*) | Mm99999915_g1 |

| **Gene** | **Forward primer sequence (5’-3’)** | **Reverse primer sequence (5’-3’)** |
| --- | --- | --- |
| *Tnf-α* | GAGGCCATTTGGGAACTTCT | TGCCTATGTCTCAGCCTCTTC |
| *Gapdh* | GAGTCAACGGATTTGGTCGT | TTGATTTTGGAGGGATCTCG |
