# Supplementary figures and images for: High Fat Diet Attenuates the Anticontractile Activity of Aortic PVAT via a Mechanism Involving AMPK and Reduced Adiponectin Secretion
Source: Front Physiol. 2018 Feb 9;9:51. doi: 10.3389/fphys.2018.00051 (PMC5812172; doi:10.3389/fphys.2018.00051)

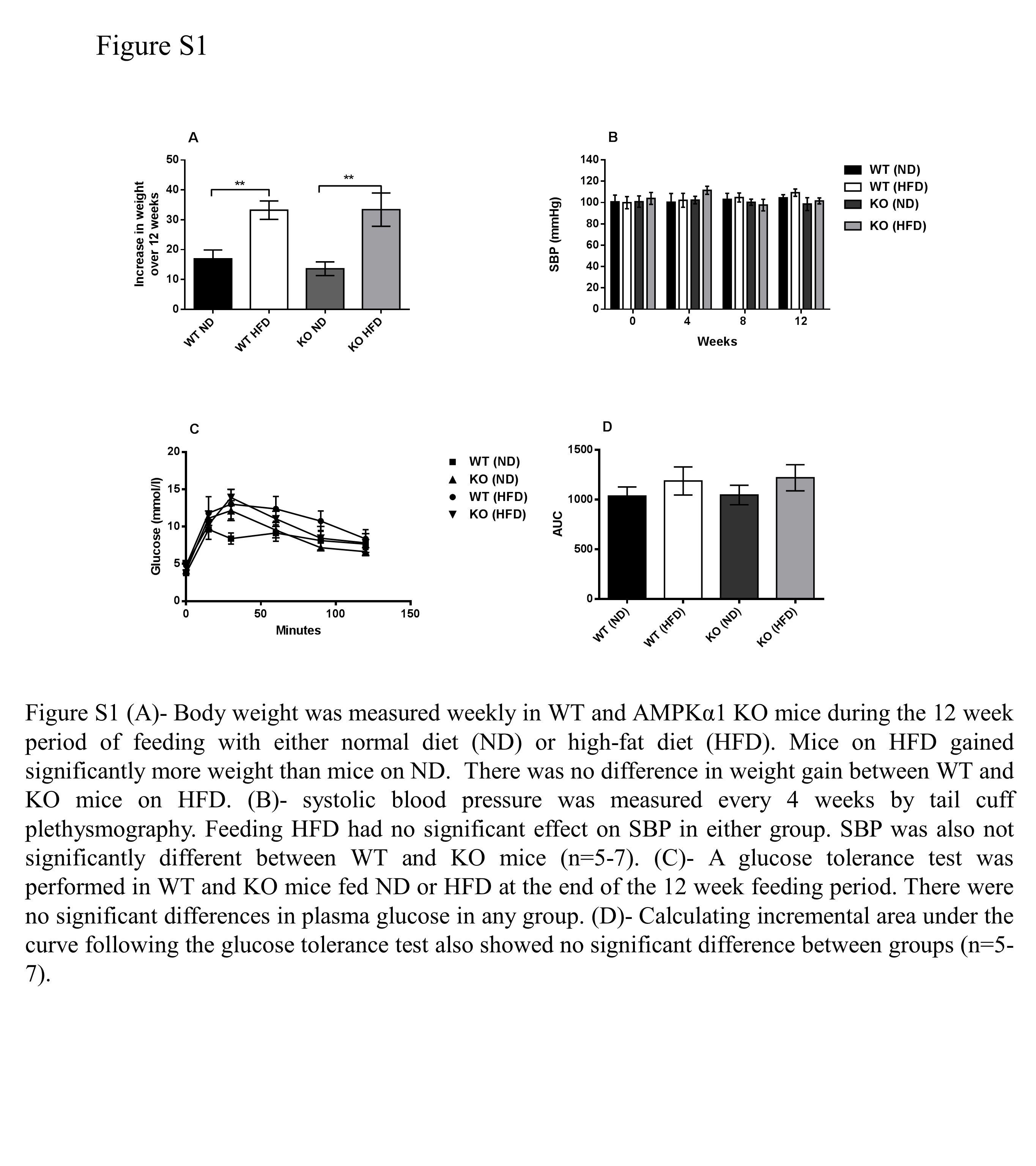

Supplement: Supplementary file 2 [file Image1.TIF]
